# Long-Term Management of Breast Cancer Bone Metastases: Two Case Reports

**DOI:** 10.1155/ijbc/9601887

**Published:** 2025-10-31

**Authors:** Xiaoting Lin, Ru Zeng

**Affiliations:** Department of Medical Oncology, The First Affiliated Hospital of Xiamen University, School of Medicine, Xiamen University, Xiamen, China

**Keywords:** bone metastases, bone-targeted agents, breast cancer, long-term use

## Abstract

Patients with advanced HR+/HER2− breast cancer often experience prolonged disease courses, with bone being a dominant site of metastasis. Bone-targeted agents (BTAs) are recommended to reduce the risk of skeletal-related events (SREs), yet most clinical trials report follow-up durations of less than 2 years. Here, we present two cases of patients with breast cancer and bone metastases who received continuous BTA therapy for 8 and 10 years, respectively, in the context of ongoing antitumor treatment. Neither patient developed SREs during follow-up. The two cases demonstrated good tolerabilit during long-term treatment, though broader conclusions regarding safety require further investigation.

## 1. Background

Bone is the most common site of metastasis in breast cancer, affecting approximately 65%–75% of patients with advanced disease [1]. Bone metastases often lead to skeletal-related events (SREs), including pathological fractures, spinal cord compression, radiation therapy to bone, and surgery to bone, each of which can significantly impair patients' quality of life and survival. Advances in antitumor therapies have extended survival time, especially for those with hormone receptor-positive/human epidermal growth factor receptor 2-negative (HR+/HER2−)breast cancer. CDK4/6 inhibitors combined with endocrine therapy have significantly improved the prognosis of patients with HR+/HER2− advanced breast cancer, extending progression-free survival (PFS) beyond 2 years and overall survival (OS) to over 5 years [2]. In patients with bone-only metastases, OS was reported to be 63.5 months in the PALOMA-2 trial [3] and 72.6 months in the MONALEESA-2 trial [4]. Long-term management of patients with breast cancer and bone metastases is a common clinical issue, however, large-scale clinical studies on bone-targeted agents (BTAs) seldom extend follow-up beyond 2 years. This article presents two cases of breast cancer with bone metastases, with a particular focus on long-term management strategies.

## 2. Methods

Two patients with advanced HR+/HER2− breast cancer and bone metastases were retrospectively selected from patients treated at The First Affiliated Hospital of Xiamen University between January 2022 and May 2024. The inclusion criterion was continuous treatment with BTAs for more than 5 years during the course of disease management, with complete follow-up records and electronic medical information available. Clinical data were collected from electronic medical records. No written consent has been obtained from the patients as there is no patient identifiable data included in this case report.

## 3. Clinical Information and Treatment Course

### 3.1. Case 1

A 45-year-old female presented with a painless lump in the right breast in April 2016. Whole-body PET-CT revealed right breast malignancy with right axillary lymph node metastasis and multiple bone metastases. Core needle biopsy of the right breast mass confirmed the diagnosis of infiltrating lobular carcinoma and lobular carcinoma in situ. Immunohistochemistry results were ER (80% +), PR (70% +), HER2 (2+, FISH–), and Ki-67 (20% +). Core needle biopsy of the right ilium revealed cancerous infiltration in the bone marrow. The patient was diagnosed with right breast cancer with right axillary lymph node and multiple bone metastases, cT2N1M1, Stage IV, luminal B (HER2–) subtype. The patient's systemic treatment regimen is outlined in Table 1.

As of May 2024, the patient remained stable. She had received bone-targeted therapy for a total of 97 months, including 60 months of zoledronic acid and 37 months of denosumab, with concurrent calcium and vitamin D supplementation. No treatment-related complications, such as hypocalcemia or osteonecrosis of the jaw, were observed.

### 3.2. Case 2

A 46-year-old woman was diagnosed with right breast invasive ductal carcinoma in April 2012 and underwent modified radical mastectomy. Pathology showed Stage IIA (pT1cN1M0), luminal B (HER2-negative) subtype. She received adjuvant chemotherapy, radiotherapy, and tamoxifen.

**Figure 1:**
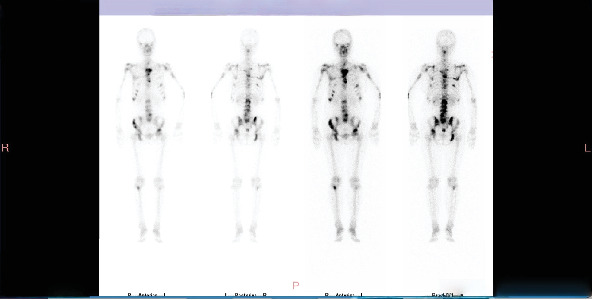
Bone scan at the second progression in July 2020 showing significant increase and intensification of bone metastases.

**Figure 2:**
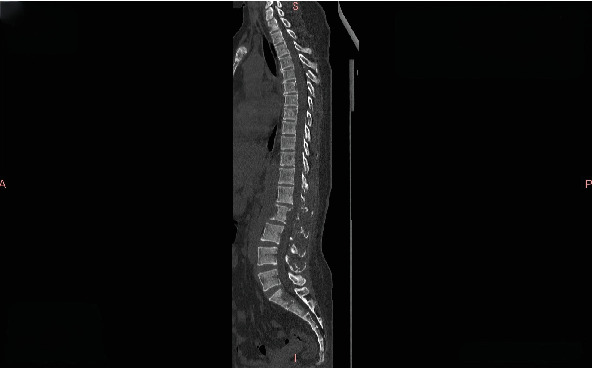
Thoracic and lumbar CT at the second progression in July 2020 showing multiple osteolytic destructions.

**Figure 3:**
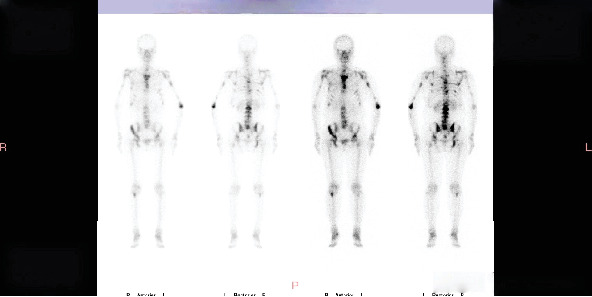
Bone scan in January 2021 showing stable bone metastases after switching antineoplastic drugs and administering denosumab treatment for 4 months.

**Figure 4:**
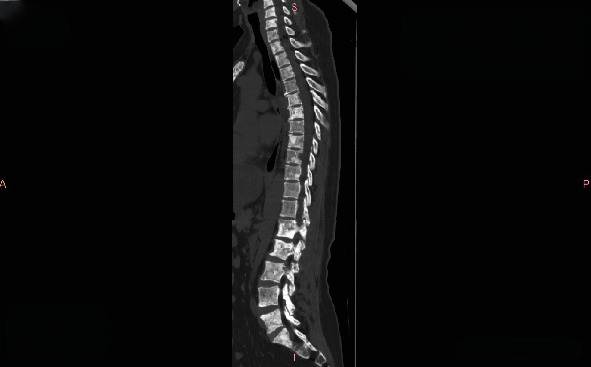
Thoracic and lumbar CT in January 2022 showing multiple bone metastases, predominantly osteoblastic lesions.

**Figure 5:**
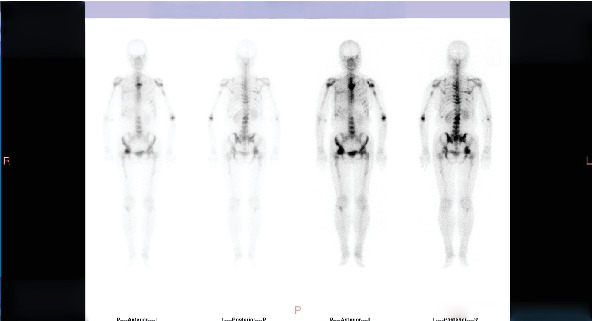
ECT at the fifth progression in February 2024 showing increased number and intensification of bone metastases.

**Figure 6:**
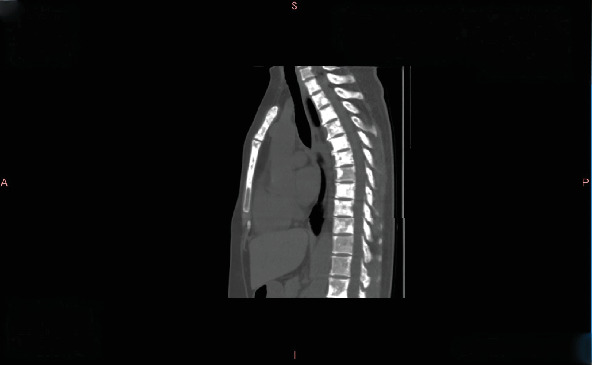
Chest CT in February 2024: multiple bone metastases similar to previous findings.

First metastasis: In April 2014, a bone scan and PET-CT confirmed bone metastasis at T12 and multiple low-metabolic nodules in both lungs. Systemic treatment after recurrence is summarized in Table 2, representative imaging findings throughout the treatment course are presented in Figures 1, 2, 3, 4, 5, and 6.

In May 2024, the patient's condition remained stable with no new progression. Up to the last follow-up, the patient has been receiving BTAs for 10 years, including 44 months of denosumab treatment.

## 4. Discussion

Patients with bone metastases who do not receive active treatment are prone to SREs such as pathological fractures and spinal cord compression, significantly impacting their quality of life and potentially being life-threatening. Breast cancer bone metastases are predominantly multiple and osteolytic, further elevating the risk of SREs. In the pre-BTA era, a study comparing pamidronate to placebo in breast cancer patients with bone metastases showed that 56% of patients in the placebo group experienced SREs [5]. In a Phase III trial comparing denosumab with zoledronic acid, 37% of enrolled patients with breast cancer and bone metastases had already experienced an SRE at baseline [6]. A retrospective observational cohort study found that patients with breast cancer and bone metastases who did not receive BTAs had a 30% risk of developing SREs within 3 months [7]. BTAs, such as bisphosphonates and denosumab, effectively reduce the risk of SREs [5, 6, 8]. According to the ESMO Bone Health Guidelines [9], BTAs should be initiated immediately upon diagnosis of bone metastases and continued throughout the disease course. The extended duration of BTAs use correlates with increased benefits for patients. A retrospective study from the United States analyzed 28,385 patients with solid tumors or multiple myeloma and bone metastases, revealing a gradual decrease in fracture incidence with increased duration of bone-protecting medication use [10].

In both cases, we reported that early initiation and continuous use of BTAs likely played a key role in preventing SREs. Case 1 did not develop any SREs during the entire disease course. In Case 2, the patient received radiotherapy to bone lesions at the time of diagnosis to prevent pathological fractures. Symptomatic skeletal event (SSE) refers to only the events accompanied by clinical symptoms, making them more relevant to patients' health-related quality of life [11]. Neither patient experienced an SSE during the course of their disease. Clinical trials have reported that, in the absence of BTAs, up to 56% of patients with breast cancer and bone metastases develop SREs [5], and even with treatment, annual SRE rates range from 30% to 40% [6]. In our cases, the absence of SREs may be attributed to effective systemic antitumor therapy that achieved durable disease control, followed by or accompanied by early and continuous use of BTAs. It is statistically plausible and reflects the potential benefit of individualized, sustained systemic and bone-directed treatment strategies.

When the patients in the cases experienced secondary progression of bone metastases, the BTAs was switched from zoledronic acid to denosumab in conjunction with a change in the antitumor treatment regimen. This resulted in a transition of multiple osteolytic lesions to osteogenic lesions, with good bone repair. Denosumab exhibits stronger inhibition of osteoclasts than zoledronic acid. As a monoclonal antibody targeting the RANK-RANKL signaling pathway, denosumab not only acts on mature osteoclasts but also inhibits their differentiation and maturation. A Phase III clinical study comparing denosumab and zoledronic acid for breast cancer bone metastases showed that denosumab significantly delayed the time to the first SRE (NR vs. 26.4 months, HR 0.82 [95% CI 0.71–0.95], *p* = 0.01) [6]. Another study found that switching to denosumab significantly reduced the risk of recurrent SREs in patients who experienced SREs during bisphosphonate treatment (HR 0.47 [95% CI 0.25–0.88], *p* = 0.019) [12].

The “flare phenomenon” refers to the radiological manifestation where existing lesions show increased tracer uptake on bone imaging, becoming more pronounced before disappearing or improving over time. In bone ECT, increased osteogenic healing leads to increased tracer uptake, resulting in increased intensity and range on ECT images. This does not indicate disease progression and may resolve or improve over time. In the most recent follow-up for these cases, the range of lesion intensity on bone ECT increased, but concurrent CT suggested similar lesions to previous scans, potentially indicating the flare phenomenon due to osteogenic repair. Further evaluation is needed based on future test results.

The prolonged survival of patients with advanced breast cancer also raises concerns about the safety of long-term use of bone-protecting agents. The question arises whether it is necessary to continue administering BTAs after 2 years of use. Given that patients with bone metastases from tumors previously had shorter survival times and large-scale clinical studies focusing on BTAs have limited data on usage exceeding 2 years, high-level evidence is currently lacking. The most significant safety concern for long-term use of BTAs is the occurrence of medication-related osteonecrosis of the jaw (MRONJ). The incidence of MRONJ is positively correlated with the duration of treatment [13]. A pooled analysis of pivotal Phase III clinical studies on denosumab (*n* = 5723) revealed a positive correlation between the incidence of MRONJ and the duration of BTAs administration, although with an overall low incidence rate of 1.8% over 0–36 months of denosumab treatment. Notably, 93% of patients who developed MRONJ in these Phase III studies had a history of tooth extraction, poor oral hygiene, or dental appliance use, with a history of tooth extraction accounting for 49% of these cases [14]. Therefore, the risk factors for MRONJ are relatively clear, and rigorous patient education and management can significantly reduce the risk of its occurrence. In both cases, careful attention was paid to oral health management, regular dental check-ups and follow-ups were conducted, and the patients were advised to avoid invasive procedures such as tooth extraction. The patients have received BTAs over several years with no severe adverse events reported. Prior evidence suggests that preventive oral care may reduce the risk of ONJ by up to 80% in patients treated with zoledronic acid [15]. Although the small sample size precludes definitive conclusions, the absence of MRONJ in these two patients may, at least in part, be attributable to regular dental monitoring and the avoidance of invasive procedures.

## Figures and Tables

**Table 1:** Disease and treatment course of Case 1.

**Treatment line**	**Antitumor treatment**	**BTA**	**Efficacy assessment**
First-line treatment (Apr. 2016–Mar. 2021)	1. Systemic chemotherapy with epirubicin and paclitaxel for 6 cycles, followed by leuprolide plus tamoxifen. Ovarian function suppression (GnRHa followed by surgical oophorectomy) 2. Modified radical mastectomy of the right breast (to relieve local symptoms)	Zoledronic acid 4 mg Q4W	Partial response

First progression: In March 2021, PET-CT showed a slight decrease in the metabolic activity of the bone metastases. However, the extent of bone metastases had significantly increased. The pathological examination of the right iliac bone biopsy was consistent with metastatic lobular carcinoma of the breast. Immunohistochemistry results: ER (60% weak–moderate +), PR (–), HER2 (1+), Ki-67 (15% +).			

Second-line treatment (Apr. 2021–Sep. 2022)	Fulvestrant + palbociclib	Denosumab 120 mg Q4W	Stable disease

Progression: In September 2022, PET-CT and biopsy confirmed liver metastasis. The metabolic activity of the original bone metastases throughout the body decreased after switching to denosumab treatment.			

Third-line treatment (Oct. 2022–May 2023)	Participation in clinical trial: Treatment with recombinant humanized HER2 monoclonal antibody-MMAE conjugate for injection (RC48-C012)	Denosumab 120 mg Q4W	Stable disease

In June 2023, the patient withdrew from the clinical trial and temporarily discontinued systemic therapy. During this treatment-free interval, in August 2023, imaging revealed a new metastasis in the left cerebellum.			

Fourth-line treatment (Aug. 2023–last follow-up)	1. Eight cycles of albumin-bound paclitaxel and then transitioned to maintenance therapy with capecitabine and anastrozole 2. Local treatment: Brain radiotherapy	Denosumab 120 mg Q4W	Stable disease

Abbreviations: BTA, bone-targeted agent; Q4W, once every 4 weeks.

**Table 2:** Disease and treatment course of Case 2.

**Treatment line**	**Antitumor treatment**	**BTA**	**Efficacy assessment**
First-line treatment (May 2014–Jun 2019)	1. Radiotherapy for bone metastases 30 Gy/10 f 2. Endocrine therapy consisted of leuprolide plus tamoxifen from May 2014 to July 2016, followed by leuprolide plus anastrozole from July 2016 to June 2019 (switch to anastrozole was made in the absence of disease progression, based on evidence supporting greater efficacy of aromatase inhibitors compared to tamoxifen when combined with ovarian function suppression)	Zoledronic acid 4 mg Q4W	Partial response

Bone metastasis progression: In May 2019, ECT revealed an increase in the number of bone metastasis sites compared to previous scans. Right iliac bone biopsy confirmed bone marrow metastatic invasive ductal carcinoma of the breast.			

Second-line treatment (Jun 2019–Jul 2020)	Leuprolide + fulvestrant	Zoledronic acid 4 mg Q4W	Stable disease

Progression: In July 2020, bone ECT indicated extensive metastatic bone tumors throughout the body with significantly increased and intensified lesions (Figure 1). Thoracic and lumbar vertebral CT showed multiple osteolytic destruction in partial thoracic vertebral bodies and their attachments, as well as some ribs and sternum (Figure 2).			

Third-line treatment (Jul 2020–Feb 2022)	1. Ovarian ablation surgery 2. Exemestane + capecitabine	Denosumab 120 mg Q4W	Stable disease

A bone scan in January 2021 showed stable disease (Figure 3). By January 2022, CT revealed multiple osteogenic metastases in the thoracic and lumbar vertebrae, ribs, sternum, scapulae, and left clavicle (Figure 4). Liver metastasis was confirmed by MRI and biopsy in February 2022.			

Fourth-line treatment (Feb 2022–Feb 2024)	Abemaciclib + fulvestrant	Denosumab 120 mg Q4W	Partial response

In March 2024, a progression of liver metastases was observed. Bone scan revealed that some of the bone metastases showed increased intensity and a larger area of concentration compared to previous scans (Figure 5). However, chest CT indicated that the multiple bone metastases remained similar to previous findings, leading to an assessment of stable bone lesions (Figure 6).			

Fifth-line treatment (Mar 2024–last follow-up)	Capecitabine + fulvestrant^a^	Denosumab 120 mg Q4W	Stable disease

Abbreviations: BTA, bone-targeted agent; Q4W, once every 4 weeks.

^a^Reintroduction of capecitabine and fulvestrant was based on prior clinical benefit, good tolerability, and limited treatment alternatives at the time. The decision was made in the context of indolent disease progression and maintained performance status.

## Data Availability

Data sharing is not applicable to this article as no new data were created or analyzed in this study.
